# Endothelin receptor B enhances liver injury and pro-inflammatory responses by increasing G-protein-coupled receptor kinase-2 expression in primary biliary cholangitis

**DOI:** 10.1038/s41598-022-21816-x

**Published:** 2022-11-17

**Authors:** Guoxin Xu, Yanping Gong, Fenying Lu, Bin Wang, Zaixing Yang, Long Chen, Jingyu Min, Cuie Cheng, Tingwang Jiang

**Affiliations:** 1 Department of Clinical Laboratory, The Affiliated Zhangjiagang Hospital of Soochow University, Zhangjiagang, 215600 China; 2 Department of Clinical Immunology, Institution of Laboratory Medicine of Changshu, Changshu, 215500 China; 3 Department of Gastroenterology, The Affiliated Changshu Hospital of Xuzhou Medical University, Suzhou, 215501 China; 4 Department of Laboratory Medicine, Huangyan Hospital of Wenzhou Medical University, Taizhou First People’s Hospital, Taizhou, 318020 China; 5 Department of Key Laboratory, The Affiliated Changshu Hospital of Xuzhou Medical University, Changshu, 215500 China

**Keywords:** Molecular biology, Zoology, Diseases

## Abstract

Severe diseases like cirrhosis and liver failure can be developed from primary biliary cholangitis (PBC). Endothelin-2 (EDN2) and endothelin receptor B (EDNRB) are related to the pathogenesis of PBC. However, the roles of EDN2 and EDNRB in PBC-related liver injury and inflammation along with molecular mechanisms are poorly defined. In this study, histopathologic alterations of liver tissues were assessed through hematoxylin–eosin staining. Alanine transaminase (ALT), alkaline phosphatase (ALP), aspartate transaminase (AST), and γ-Glutamyltranspetidase (GGT) (4 liver function indexes) serum levels were detected with corresponding activity assay kits. Also, we determined the levels of M2 subtype anti-mitochondrial antibody (AMA-M2), interferon-gamma (IFN-γ), and tumor-necrosis factor alpha (TNFα) in serum with ELISA assay. Later, RT-qPCR assay was used to measure the expression of genes at mRNA levels, while western blotting and immunohistochemical techniques were used to detect protein levels of genes. Our results showed that the liver tissues of PBC patients and mice presented with severe hepatocyte injury and inflammatory cell infiltration as well as destruction of intrahepatic small bile ducts. ALP, AST, ALT, GGT, AMA-M2, IFN-γ, and TNF-α serum levels were higher in PBC patients and mice. Besides, EDN2 and EDNRB were highly expressed in serums and livers of PBC patients and mice. EDNRB potentiated PBC-related liver injury and pro-inflammatory responses, as evidenced by observation of serious liver pathologic injury and increased serum levels of ALP, AST, ALT, AMA-M2, IFN-γ, and TNF-α in PBC mice following EDNRB overexpression. EDNRB overexpression or activation via its agonist IRL-1620 TFA triggered liver injury and pro-inflammatory responses, increased GRK2 expression and induced NF-κB expression and activation in wild-type mice. EDNRB knockdown or inhibition by Bosentan alleviated liver damage and inflammation, reduced GRK2 expression, and inhibited NF-κB in PBC mice. These findings suggested EDNRB loss or inhibition weakened liver injury and pro-inflammatory responses by down-regulating GRK2 and inhibiting the NF-κB pathway in PBC mice.

Chronic inflammatory and auto-immune cholestasis hepatic disorder like primary biliary cholangitis (PBC), (also previously termed as primary biliary cirrhosis) is portrayed by formation of granuloma, infiltration of lymphocyte and destruction of small intrahepatic bile ducts, destructive chronic non-suppurative cholangitis, presence of disease specific antimitochondrial antibody (AMA), inflammationcf6u v and liver injury^1–3^. It has been reported that PBC incidence varies from 0.84 to 2.75 per 100,000 persons, while its prevalence is approximately 1.91–40.2/100,000^4^. Moreover, increasing rates of PBC incidence and prevalence have been observed in multiple areas including Europe and Asia^4,5^. Despite the lower incidence and prevalence, PBC is a major threat to the life of patients because it can induce multiple serious diseases such as portal hypertension, cirrhosis and liver failure^3,4,6^. Currently, PBC is mainly treated with ursodeo-xycholic acid (UDCA)^7^. However, the therapeutic outcomes of UDCA are unfavorable for a substantial PBC population^7^. Thus, for improved management of PBC, an in-depth insight into mechanisms of PBC progression at molecular level.

Endothelin (ET) is an important factor in regulating cardiovascular function, plays an important role in maintaining basic vascular tone and homeostasis of the cardiovascular system, and is divided into EDN1, EDN2 and EDN3 according to different structures. EDN1 is mainly expressed in the vascular endothelium, respiratory epithelium, myocardium, fibroblasts and other cells and brain neurons. The site where EDN2 and its corresponding receptors bind Expressed and functioning in ovarian and intestinal epithelial cells^8,9^. EDN3 is mainly expressed in the nervous system and is involved in the development of neural crest cells. EDN3 and is necessary for the formation of nerves in the intestinal nerve and melanocytes. Endothelin receptor (EDNR) is obtained by its effect with endothelin through the G protein-coupled endothelin receptor pathway. At present, only two receptors associated with endothelin, EDNRA and EDNRB, have been found in mammals, and previous studies have found that EDNRA is mainly found in cells such as heart, cerebrovascular and aortic smooth muscle cells (VSMC), to promote the division and proliferation of cells, vasoconstriction and tissue fibrosis and other effects. While EDNRB is able to indirectly cause vasodilation, primarily, by acting with endothelin Promote vasodilation by stimulating cells to release NO with PGI2. Dysregulation of endothelins and endothelin receptor systems is closely linked with pathogenesis of multiple diseases namely autoimmune disorders (*e.g.* rheumatoid arthritis, lupus and multiple sclerosis)^8,10^. Recent studies also suggested that endothelins and endothelin receptors might influence liver function and regulate liver disease progression^11–13^. For instance, the blockade of EDNRA by BQ-123 notably reduced the portal pressure in patients with cirrhosis^14^. The introduction of EDNRA and EDNRB antagonists BQ-123 and BQ-788 could markedly increase the expression levels of fibrosis marker, collagen 1A1 and three cytokines of proinflammation (i.e. IL-1β, IL-6 and TNFα,) in Hep3B cells^15^.

Moreover, earlier works have posited that endothelins and endothelin receptors may be implicated in biliary cirrhosis^16–18^. Exemplary, Kojima et al*.* evidenced that EDN1 and ENDRB expression levels and portal pressure were up-regulated in liver tissues of PBC rats, wherein the model was induced by bile duct ligation, amid ENDRA level demonstrating no notable alteration after bile duct ligation^17^. The intraportal injection of EDN1 and ENDRB agonist sarafotoxin 6c could improve the portal pressure of rats^17^. Additionally, Dimoulios et al*.* observed higher serum levels of endothelin-2 (EDN2) in PBC subjects compared to their healthy counterparts^19^. Moreover, EDN2 serum level was notably reduced in PBC patients after UDCA treatment^19^. These data suggested that EDN2 and ENDRB may play crucial roles in PBC development.

In this text, we further investigated roles and molecular mechanistic action of EDN2 and ENDRB in PBC.

## Materials and methods

### Clinical sample collection

We collected liver tissues from 5 PBC patients and 5 healthy individuals (who were matched in terms of age and sex) through needle biopsy between March and May 2021. Serum specimens were isolated from blood samples of 10 PBC patients and 10 healthy volunteers (who were matched in terms of age and sex). Liver tissues and blood samples were collected at the Affiliated Changshu Hospital of Xuzhou Medical University, China and the Affiliated Zhangjiagang Hospital of Soochow University, China, respectively (the ratio of samples collected = 2:3). The study was performed in accordance with the Declaration of Helsinki and was approved by the ethics committee of Affiliated Changshu Hospital of Xuzhou Medical University and the Affiliated Zhangjiagang Hospital of Soochow University. All methods were conducted in accordance with relevant guidelines and regulations. Also, all the subjects gave their written informed consents.

### Real-time PCR

The mRNA sequencing analysis for the liver tissues of 5 PBC patients and 5 healthy individuals (who were matched in terms of age and sex) was performed by Novogene Co., Ltd. (Beijing, China). Isolation of total RNA from liver tissues and samples of serum was accomplished with the Trizol reagent. Later, we conducted reverse transcription reaction with the M-MLV reverse transcriptase to synthesize cDNA first-strand. Using Fast SYBR Green Master-mix , we performed quantitative PCR, wherein the internal control was β-actin. Later on, we analyzed relative expression with ^2−ΔΔCt^ method. Ct values are counted using excel.

### Assaying with western blotting

Extraction of protein from serum and liver samples was accomplished with RIPA buffer (Beyotime Biotech, Shanghai-China) comprising inhibitors of phosphatase and protease (Thermo-Fisher Scientific). Pierce-BCA protein assay kit (Thermo-Fisher Scientific) was used to analyze protein content. Separation of protein samples (40 μg/lane, equal amounts) through SDS-PAGE was carried out prior to eletro-transfer onto membranes of poly-vinylidene fluoride (Millipore, Bedford, MA-USA). Blocking of non-specific signals on the membranes was accomplished with skimmed milk (5%). Next, incubation of proteins was done with corresponding antibodies, viz., primary and secondary labeled with horseradish peroxidase. We developed the protein bands with substrate of Pierce ECL western blotting (Thermo-Fisher Scientific).

### Biochemical index detection

We measured serum levels of alanine transaminase (ALT), alkaline phosphatase (ALP), aspartate transaminase (AST), and γ-glutamyltranspetidase (GGT) by corresponding kits (Elabscience, Wuhan-China). Human and mouse interferon gamma (IFN-γ) serum levels were examined by matching ELISA kits ((Elabscience, Wuhan-China). Serum levels of human and mouse tumor-necrosis factor alpha (TNFα) were detected with corresponding ELISA kits ((Elabscience, Wuhan-China). AMA-M2 serum level was measured via anti-AMA-M2 ELISA kit (Cusabio, Wuhan-China).

### HE staining

HE staining of patients with PBC and normal liver tissue. Dewaxing and rehydration of paraffinized embedded sections was accomplished with ethanol and xylene. Later, HE staining of the sections was carried out. We imaged the sections after dehydration and sealing treatments. Inflamed cell infiltration around the interlobular bile ducts, hepatocellular edema/necrosis/fibrosis, and decreased number of bile ducts in the liver were observed.

### Immunohistochemical (IHC) analysis

Fix liver tissue for 15 min using 4% paraformaldehyde, wash, permeabilize cells with PBS supplemented with 0.2% Triton-X, block with 5% serum in PBS, and incubate overnight at 4 °C with FITC-coupled anti-EDNRB, GRK2, NF-κB, IKKα, IKKβ, p-NF-κB, p-IKKα and p-IKKβ monoantigen antibodies. After washing, install the unit with Vectorield installation media with DAPI. Photographed under Olympus laser confocal microscopy and ordinary fluorescence microscope.

### Mice experiments

Conduct of mice experiments was carried out based on guidelines of the Institutional Animal Care and Use Committee of Xuzhou Medical University. Bangyao Biotechnology Co., Ltd supplied IL-2Rα −/− and wild type mice (6–8 weeks old). Adenoviruses carrying EDNRB knockdown fragment (Ad-shEDNRB) or EDNRB full-length CDS (Ad-EDNRB) were customized from Bangyao Biotechnology Co., Ltd. Normal saline or adenoviruses were injected into mice via the tail vein. EDNRB−/− mice were generated using the CRISPR-Cas9 technology by Bangyao Biotechnology Co., Ltd. EDNRB−/− IL-2Rα −/− mice were established through hybridization. IRL-1620 TFA or Bosentan was injected into mice.

### Statistical analysis

Analysis of data was accomplished with Graphpad software (Version 7, Graphpad, San Diego, CA-USA). Outcomes were displayed as mean and standard deviation. Analysis of statistical difference within groups was carried out with Student’s t-test. Comparison of difference within groups was accomplished with one-way ANOVA and Tukey’s test. Statistically, we defined significant difference as significant at *p*-value ˂ 0.05.

### Ethical approval

Animal experiments were approved by the Institutional Animal Care and Use Committee of Xuzhou Medical University [No. L20210226327] and performed in accordance with ARRIVE guidelines for the Care and Use of Laboratory Animals.

Human experiments were performed in accordance with the Declaration of Helsinki and was approved by the ethics committee of the Affiliated Changshu Hospital of Xuzhou Medical University [No. 2020-KY-001] and the Affiliated Zhangjiagang Hospital of Soochow University [No. 20200101]. All methods were conducted in accordance with relevant guidelines and regulations and each participant was informed their right to have their information kept confidential.

## Results

### Histopathological and serum biochemical index analysis of PBC patients

**Figure 1:**
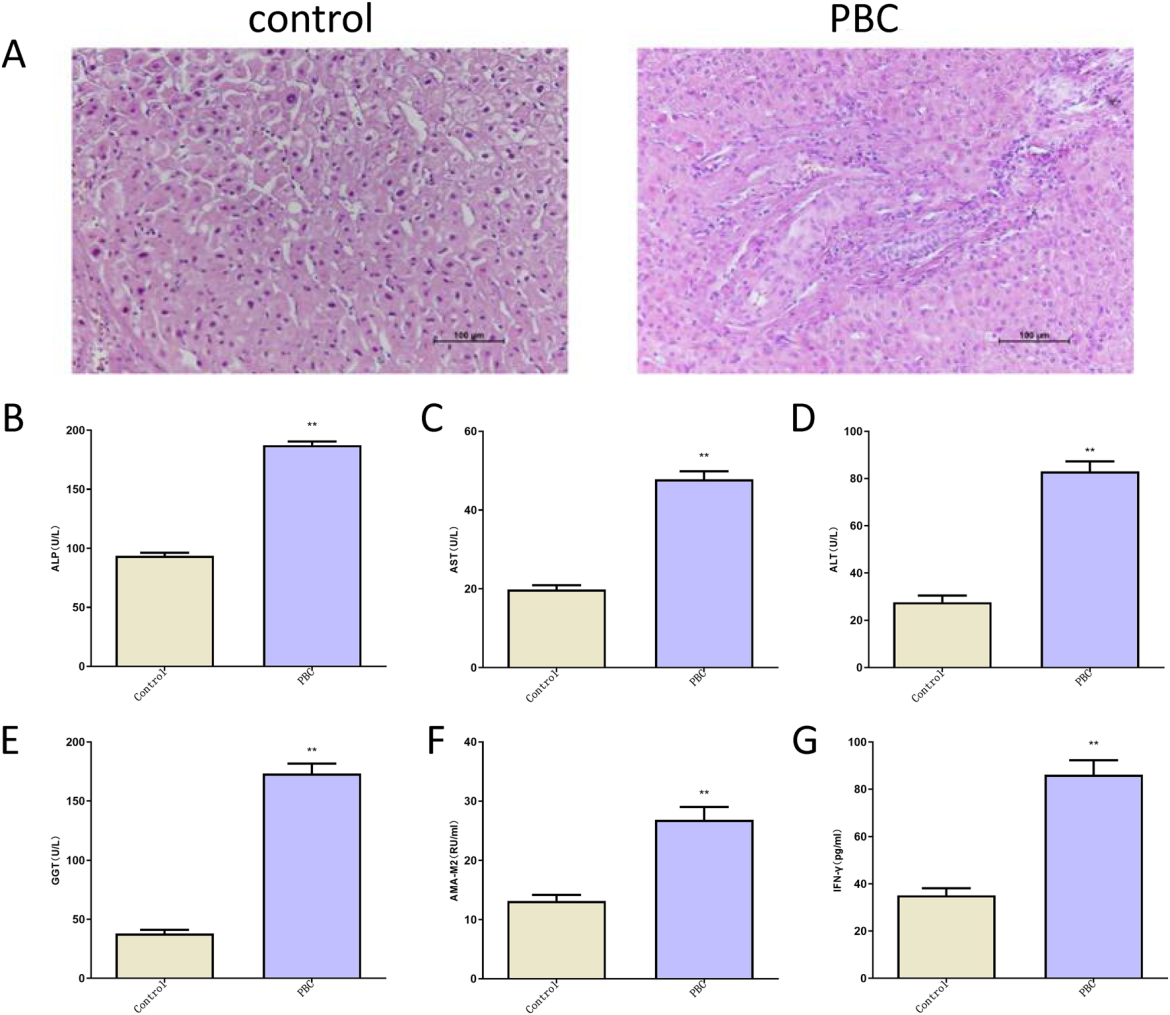
Histopathological and serum biochemical index analysis of PBC patients. (**A**) HE staining examination of liver tissues obtained from PBC subjects and healthy volunteers. (**B–G**) The levels of ALP, AST, ALT, GGT, AMA-M2, and IFN-γ in samples of PBC patients’ sera (n = 10) and healthy volunteers (n = 10) were measured by ELISA assay.

To explore pathological changes in liver tissue in patients with PBC, we detected morphological differences by HE staining (Fig. 1A). HE staining analysis showed that liver tissues of PBC patients presented notably pathological alterations such as hepatocyte edema/necrosis/fibrosis, and significantly damaged (Fig. 1E). Also, levels of ALT, ALP, AST and GGT (4 liver function indexes) in serum were markedly increased in PBC patients comparable to healthy control group (Fig. 1B–E). Moreover, AMA-M2, a diagnostic marker for PBC was highly expressed in serums of PBC patients (Fig. 1F). Additionally, increased pro-inflammatory cytokine IFN-γ level in serum was observed in PBC patients compared to healthy individuals (Fig. 1G).

### EDN2 and EDNRB expression levels were notably up-regulated in the liver tissues and serum samples of PBC patients relative to the healthy control group

**Figure 2:**
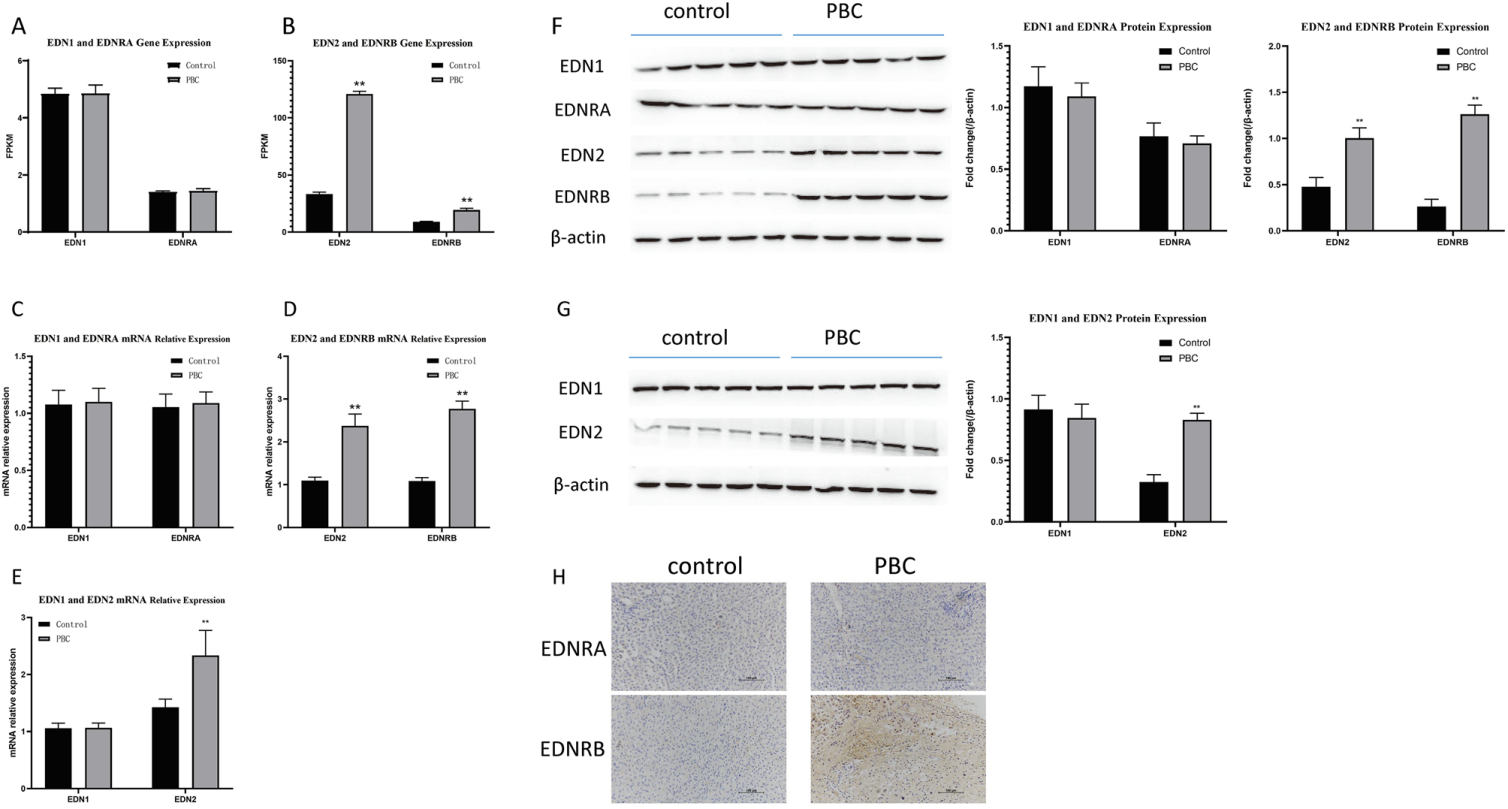
EDN2 and EDNRB expression levels were notably up-regulated in samples of liver tissues and sera of PBC patients relative to healthy control group. (**A** and **B**) RNA-seq outcomes of EDN1, EDNRA, EDN2, and EDNRB in liver tissues of patients with PBC (n = 5) and healthy volunteers (n = 5). (**C** and **D**) RT-qPCR technique was used to measure levels of EDN1, EDNRA, EDN2, and EDNRB mRNAs in liver tissues of patients with PBC (n = 5) and healthy volunteers (n = 5). (**E**) Determination of EDN1 and EDN2 mRNAs in sera of PBC patients (n = 10) and healthy volunteers (n = 10) was accomplished with RT-qPCR technique. (**F**) Measurement of EDN1, EDNRA, EDN2, and EDNRB proteins in liver tissues of patients with PBC (n = 5) and healthy volunteers (n = 5) was carried out with western blotting technique. (**G**) We examined the protein levels of EDN1 and EDN2 in serum samples of 5 random PBC patients and 5 random healthy volunteers with western blotting technique. (**H**) Illustrative IHC images of EDNRA and EDNRB in liver tissues of healthy people and patients with PBC.

RNA-seq outcomes demonstrated no notable difference in EDN1 and EDNRA expression levels in liver tissues of PBC patients (n = 5) compared to healthy control group (n = 5) (Fig. 2A). However, EDN2 and EDNRB expression levels increased markedly in liver tissues of the patients (n = 5) comparable to healthy control group (n = 5) (Fig. 2B). Through RT-qPCR analysis, we further showed obvious up-regulation of EDN2 and EDNRB mRNA levels in hepatic tissues of PBC patients (n = 5) compared to healthy control samples (n = 5) (Fig. 2C). Moreover, EDN2 mRNA level was higher in serum samples of patients with PBC (n = 10) compared to healthy counterparts (n = 10) (Fig. 2E). Through western blotting technique, we observed obvious increased levels of EDN2 and EDNRB protein in liver samples of PBC patients (n = 5) in comparison with liver tissues of healthy people (n = 5) (Fig. 2F). Furthermore, increased EDN2 protein expression was observed in serum samples of PBC patients (n = 5) relative to the healthy control group (n = 5) (Fig. 2G). Comparable to healthy control group, high expression of EDNRB was validated with IHC in liver samples of PBC patients (Fig. 2H). Nevertheless, levels of EDN1 and EDNRA mRNA and protein were not obviously altered in samples of hepatic tissues and sera of PBC subjects relative to healthy control group (Fig. 2D–H).

### Histopathological and serum biochemical index analysis of PBC mice

**Figure 3:**
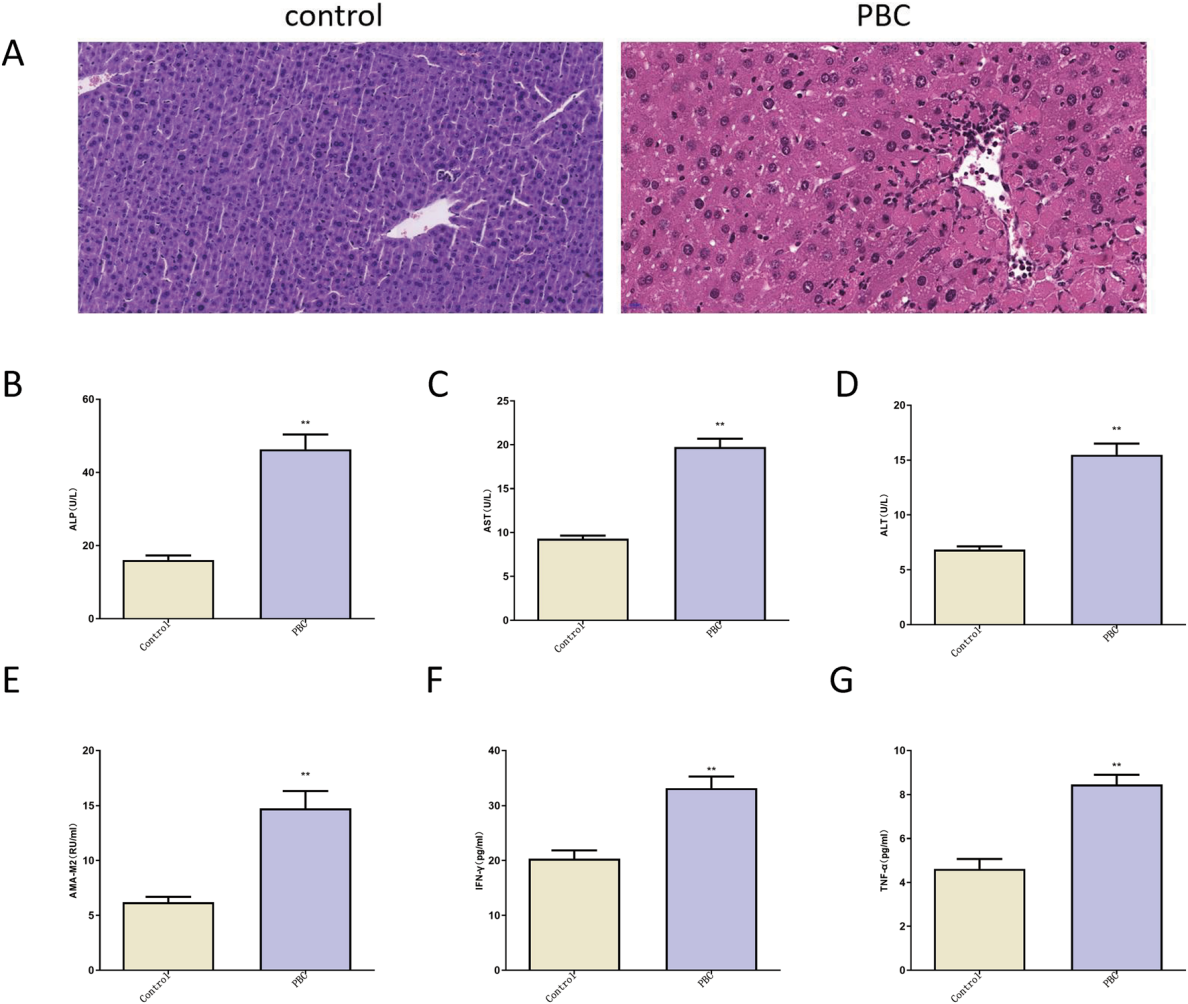
Histopathological and serum biochemical index analysis of PBC mice. (**A**) HE staining examination of liver tissues obtained from PBC mice (n = 10) and wild type mice (n = 10). (**B**–**G**) Levels of ALP, AST, ALT, AMA-M2, IFN-γ, and TNF-α in serum samples of PBC mice (n = 10) and wild type mice (n = 10) were measured by corresponding kits and ELISA assay.

IL-2Rα -/- mice are commonly used PBC spontaneous models^20,21^. Herein, an obvious hepatocyte edema, more inflammatory cell infiltration around interlobular bile ducts, and disorganized hepatocytes were observed in liver tissues of IL2Rα-depleted mice than in the control mice (Fig. 3A). Moreover, up-regulation of serum levels of ALP, AST, ALT, AMA-M2, IFN-γ and TNFα was noticeably observed in IL2Rα-depleted mice compared to control mice (Fig. 3B–G). These data suggested successful establishment of IL-2Rα-/- PBC mouse models.

### High expression of EDN2 and EDNRB in the liver tissues and sera of PBC mice and wild type mice

**Figure 4:**
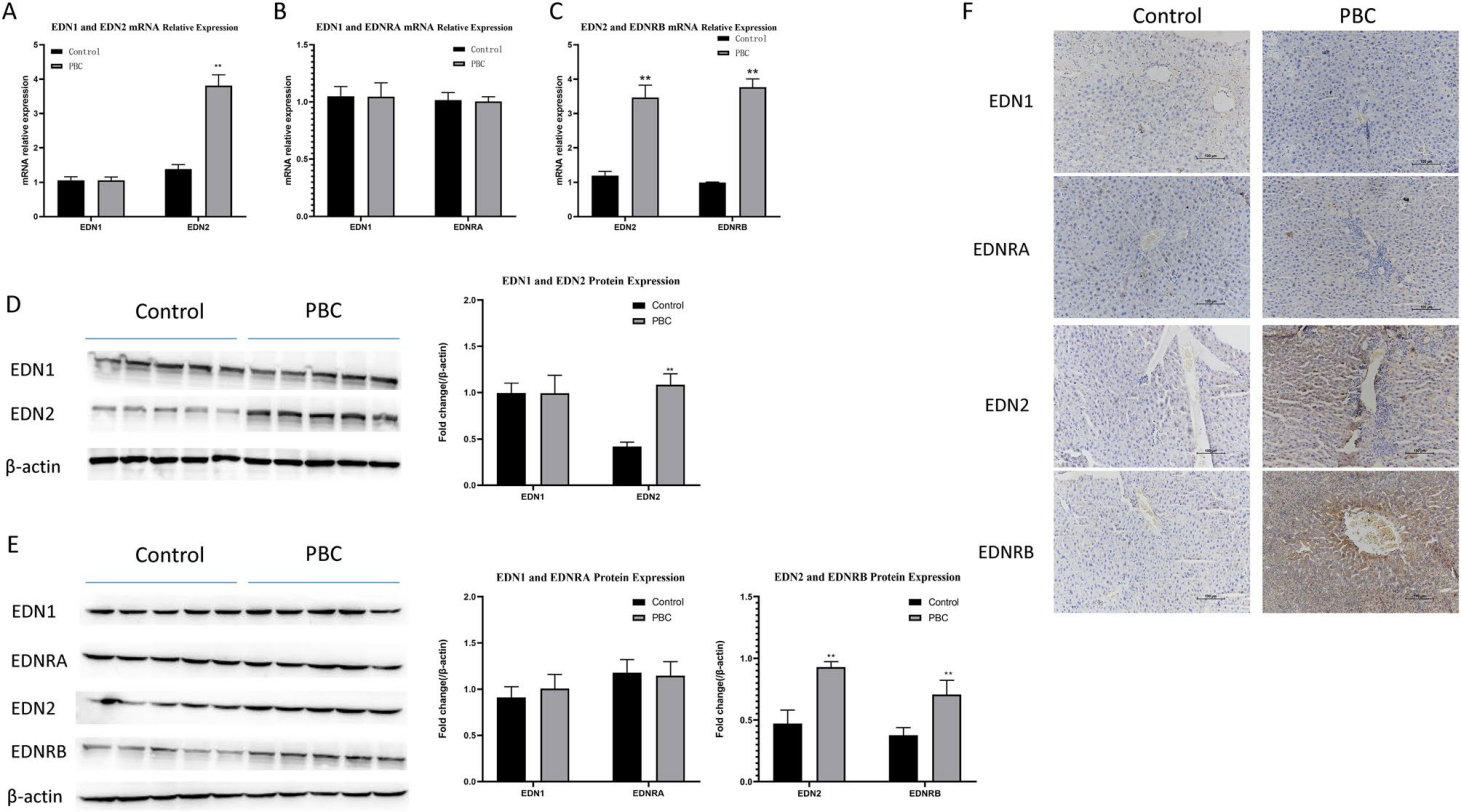
High expression of EDN2 and EDNRB in liver tissues and sera of PBC mice and wild type mice. (**A**) RT-qPCR technique was used to determine mRNA levels of EDN1 and EDN2 in sera of PBC mice (n = 10) and wild type mice (n = 10). (**B** and **C**) Measurement of EDN1, EDNRA, EDN2, and EDNRB mRNAs in liver tissues of BC mice (n = 10) and wild type mice (n = 10) was accomplished with RT-qPCR technique. (**D**) Levels of EDN1 and EDN2 proteins in serum samples of 5 random PBC mice and 5 random wild type mice were examined with western blotting technique. (**E**) Determination of EDN1, EDNRA, EDN2 and EDNRB proteins in liver tissues of 5 random PBC mice and 5 random wild type mice was accomplished with western blotting assay. (**F**) Illustrative IHC images of EDN1, EDNRA, EDN2, and EDNRB in liver tissues of PBC mice and wild type mice.

The detection of EDN2 levels by Western Blotting and RT-PCR showed that the mRNA and protein expression levels of EDN2 increased in the serum of PBC mice (Fig. 4A,D). Also, RT-PCR, western blotting and IHC analysis demonstrated markedly increased levels of EDN2 and EDNRB expressions in liver tissues of PBC mice comparable to control (Fig. 4C,E,F). Similar to clinical outcomes, there was no obvious difference in EDN1 and EDNRA expression between the PBC mouse and wild type mice groups (Fig. 4A,B,D–F).

### Effects of EDNRB overexpression or knockdown on PBC development

**Figure 5:**
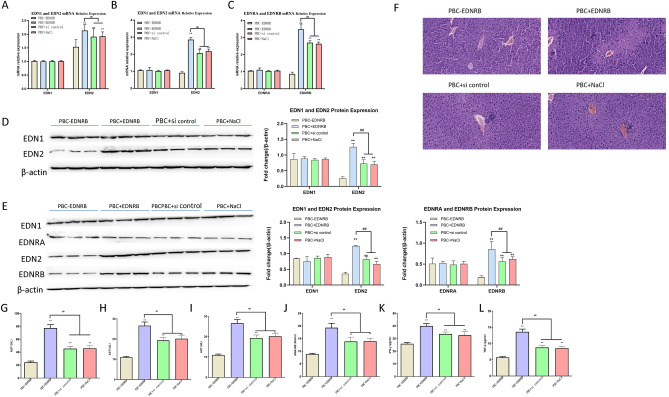
Effects of EDNRB overexpression or knockdown on PBC development. (**A**) Determination of EDN1 and EDN2 mRNAs was accomplished with RT-qPCR technique in sera of PBC mice injected with normal saline (PBC + NaCl), control adenoviruses (PBC + Si Control), Ad-shEDNRB adenoviruses (PBC-EDNRB), and Ad-EDNRB adenoviruses (PBC + EDNRB). Each group contained 10 mice. (**B** and **C**) RT-qPCR assay was used to measure levels of EDN1, EDNRA, EDN2, and EDNRB mRNAs in liver tissues of PBC mice injected with normal saline, control adenoviruses, Ad-shEDNRB adenoviruses and Ad-EDNRB adenoviruses. Each group contained 10 mice. (**D**) Measurement of EDN1 and EDN2 expression at protein levels was examined with western blotting assay in serum samples of PBC mice injected with normal saline, control adenoviruses, Ad-shEDNRB adenoviruses, and Ad-EDNRB adenoviruses. Each group contained 3 mice. (**E**) Levels of EDN1, EDNRA, EDN2 and EDNRB proteins were determined with western blotting technique in liver tissues of PBC mice injected with normal saline, control adenoviruses, Ad-shEDNRB adenoviruses, and Ad-EDNRB adenoviruses. Each group contained 3 mice. (**F**) HE staining of liver tissues of mice injected with normal saline, control adenoviruses, Ad-shEDNRB adenoviruses, and Ad-EDNRB adenoviruses. (**G**–**L**) Levels of ALP, AST, ALT, AMA-M2, IFN-γ and TNFα in serum samples of PBC mice injected with normal saline, control adenoviruses, Ad-shEDNRB adenoviruses, and Ad-EDNRB adenoviruses were measured with corresponding kits and ELISA assay.

To further investigate the functions of EDNRB in PBC progression, adenoviruses carrying EDNRB knockdown fragment (Ad-shEDNRB) and EDNRB full-length CDS (Ad-EDNRB) were produced. Through western blotting and RT-PCR analyses, we showed marked elevation of EDNRB mRNA and protein in samples of sera and liver tissues of PBC mice following the injection of Ad-EDNRB adenoviruses (Fig. 5A–E). Conversely, the inoculation of Ad-shEDNRB adenoviral particles resulted in obvious decreased mRNA and protein of EDNRB in samples of sera and liver tissues of PBC mice (Fig. 5A–E). Moreover, EDNRB up-regulation or down-regulation did not influence EDN1 and EDNRA expression in PBC mice (Fig. 5A–E). HE analysis showed that liver tissues of PBC mice after EDNRB overexpression displayed more severe liver injury (e.g. fibrosis) and more immune cell infiltration relative to control groups, while EDNRB-depleted mice presented less liver pathological injury (Fig. 5F). Moreover, enforced expression of EDNRB triggered a notable increased levels of ALT, ALP, AST, AMA-M2, IFN-γ, and TNF-α in sera of PBC mice (Fig. 5G–L). Conversely, there was a marked down-regulation in serum levels of ALP, AST, ALT, AMA-M2, IFN-γ and TNF-α in PBC mice following EDNRB knockdown (Fig. 5G–L). These data suggested that EDNRB might aggravate PBC.

### EDNRB overexpression or activation induced liver damage by increasing GRK2 expression and activating NF-κB pathway in wild-type mice

**Figure 6:**
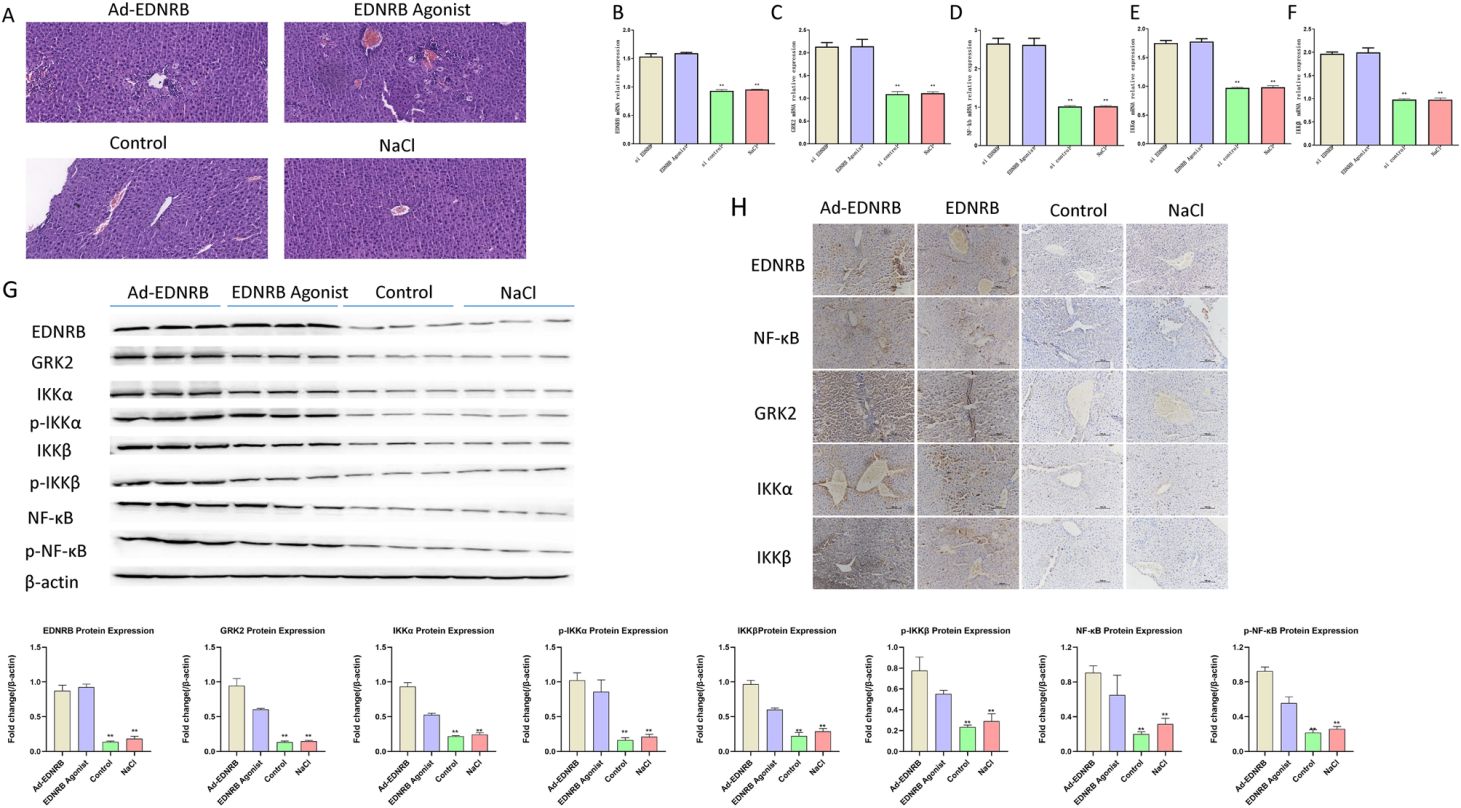
EDNRB overexpression or activation induced liver damage by increasing GRK2 expression and activating NF-κB pathway in wild type mice. (**A**) HE staining of liver tissues of mice injected with normal saline (NaCl), control adenoviruses (Control), Ad-EDNRB adenoviruses (Ad-EDNRB), or IRL-1620 TFA (EDNRB agonist) Each group contained 10 mice.. (**B**–**F**) The mRNA levels of EDNRB, GRK2, NF-κB, IKKα and IKKβ were measured with RT-qPCR assay in liver samples of mice injected with normal saline, control adenoviruses, Ad-EDNRB adenoviruses, or IRL-1620 TFA. (**G**) Western blotting technique was used to detect protein levels of EDNRB, GRK2, NF-κB, IKKα, IKKβ, p-NF-κB, p-IKKα, and p-IKKβ in liver tissues of mice injected with normal saline, control adenoviruses, Ad-EDNRB adenoviruses, or IRL-1620 TFA. (**H**) Levels of EDNRB, GRK2, NF-κB, IKKα, and IKKβ proteins were measured with IHC assay in liver tissues of mice injected with normal saline, control adenoviruses, Ad-EDNRB adenoviruses, or IRL-1620 TFA.

The experiment was divided into 4 groups: Ad-EDNRB adenoviruses (Ad-EDNRB), EDNRB agonist IRL-1620 TFA (EDNRB agonist), normal saline (NaCl), control adenoviruses (Control). HE outcomes showed that wild type mice after treatment with Ad-EDNRB adenoviral particles or EDNRB agonist IRL-1620 TFA presented serious liver pathologic injury such as more inflammatory infiltration, hepatocyte necrosis and edema (Fig. 6A). Western blotting, RT-qPCR and IHC techniques depicted obviously increased levels of EDNRB mRNA and protein in liver tissues of wild type mice following injection of Ad-EDNRB adenoviral particles or the stimulation of EDNRB agonist IRL-1620 TFA (Figs. 6B, G, H). Moreover, EDNRB overexpression or IRL-1620 TFA stimulation facilitated expression of GRK2, NF-κB, IKKα, and IKKβ mRNAs and proteins, which triggered a noticeable increase in levels of p-IKKα, p-IKKβ and p-NF-κB proteins in liver tissues of wild type mice (Figs. 6C–H). These data suggested that EDNRB overexpression or activation induced liver pathologic damage in wild type mice by increasing GRK2 expression and activating NF-κB pathway.

**Figure 7:**
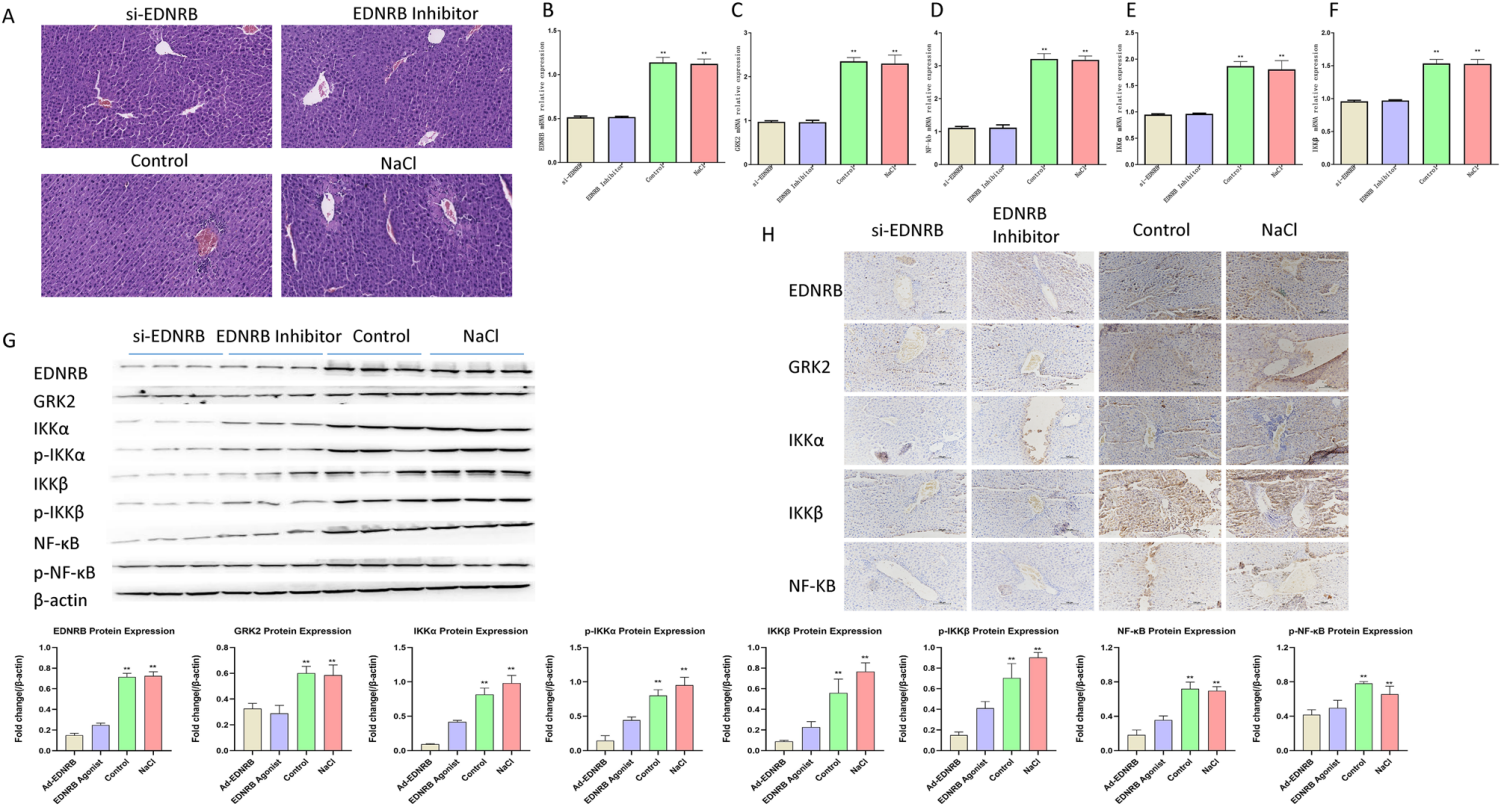
EDNRB knockdown or inhibition alleviated liver pathologic damage by suppressing GRK2 expression and inactivating NF-κB pathway in PBC mice. (**A**) HE staining of liver tissues of mice injected with normal saline (NaCl), control adenoviruses (Control), Ad-shEDNRB adenoviruses (Si-EDNRB), or EDNRB inhibitor Bosentan (EDNRB inhibitor). Each group contained 10 mice. (**B**–**F**) The mRNA levels of EDNRB, GRK2, NF-κB, IKKα, and IKKβ were measured with RT-qPCR assay in liver samples of mice injected with normal saline, control adenoviruses, Ad-shEDNRB adenoviruses, or EDNRB inhibitor Bosentan. (**G**) The protein levels of EDNRB, GRK2, NF-κB, IKKα, IKKβ, p-NF-κB, p-IKKα and p-IKKβ were detected via western blotting technique in liver tissues of mice injected with normal saline, control adenoviruses, Ad-shEDNRB adenoviruses, or EDNRB inhibitor Bosentan. (**H**) Levels of EDNRB, GRK2, NF-κB, IKKα, and IKKβ proteins were measured through IHC assay in liver tissues of mice injected with normal saline, control adenoviruses, Ad-shEDNRB adenoviruses, or EDNRB inhibitor Bosentan.

### EDNRB knockdown or inhibition alleviated liver pathologic damage by suppressing GRK2 expression and inactivating NF-κB pathway in PBC mice

Ad-shEDNRB adenoviruses (Si-EDNRB), EDBRB inhibition Bosentan (EDNRB inhibitor), normal saline (NaCl), control adenoviruses (Control) treated with EDNRB knockdown mice. Histopathologic analysis showed that EDBRB depletion or inhibition alleviated PBC-induced liver injury, inflammatory infiltration, and cell necrosis (Fig. 7A). Furthermore, we showed through western blotting, RT-qPCR and IHC techniques that EDBRB loss or inhibition resulted in obvious reduced levels of EDNRB, GRK2, NF-κB, IKKα, IKKβ, p-NF-κB, p-IKKα and p-IKKβ in liver tissues of PBC mice (Fig. 7B–H).

## Discussion

Herein, we have demonstrated that liver tissues of PBC patients and PBC mice presented with notable histopathological injury such as increased inflammatory cell infiltration around interlobular bile ducts, hepatocyte edema/necrosis/fibrosis, and reduced number of intrahepatic small bile ducts. Also, higher serum levels of liver dysfunction indexes (i.e. ALP, AST, ALT, and GGT), immunological index AMA-M2, and inflammatory factors IFN-γ and TNF-α were noticed in PBC patients relative to the healthy control group. These histopathological, biochemical, and immunopathologic indexes are frequently used as proxies for the diagnosis of PBC^22,23^. Moreover, RNA-seq, RT-qPCR, western blot, and IHC assays showed that EDN2 and ENDRB were highly expressed in liver tissues and serum samples of PBC patients and PBC mice. The high expression of EDN2 in serum samples of PBC patients also has been identified in a previous report^19^. There was no notable difference in endothelin-1 serum level between the PBC group and the healthy control group^19^.

Additionally, we demonstrated that the knockdown of ENDRB or inhibition of ENDRB by Bosentan alleviated the pathological injury of the liver tissues and reduced the serum levels of ALP, AST, ALT, AMA-M2, IFN-γ, and TNF-α in PBC mouse models, suggesting the ameliorative effect of ENDRB loss on PBC. Conversely, ENDRB overexpression or activation by its agonist IRL-1620 TFA further potentiated PBC-induced liver pathological injury and increased PBC-induced expression of the above-mentioned liver dysfunction, immunological, and inflammatory indexes.

ENDRA and ENDRB are two members of G protein-coupled receptors (GPCRs)^24^. It has been reported that there was a complex interaction between GPCRs and GRK2. For instance, some studies showed that GRK2 could control desensitization and internalization of multiple GPCRs and regulate GPCR related signaling transduction pathways including endothelin receptor signaling^25–27^. However, a prior study suggested that the activation of beta2-adrenergic receptors (GPCRs) can modulate GRK2 ubiquitination and proteolysis, resulting in the rapid degradation^28^. Moreover, G protein-coupled receptor kinase 2 (GRK2) has been found to be involved in the regulation of multiple biological processes such as angiogenesis, metabolism, vasodilatation, immunity, and inflammation^26,29^. Moreover, GRK2 is implicated in the progression and pathogenesis of multiple diseases including autoimmune, fibrotic, and liver disorders^27,30,31^. Hence, we further investigated the effect of ENDRB overexpression, activation, knockdown, or inhibition on GRK2 expression in liver tissues of PBC mice. Results showed that ENDRB overexpression or ENDRB agonist IRL-1620 TFA injection facilitated GRK2 expression in liver tissues of PBC mice. Inversely, ENDRB loss or blockade reduced GRK2 expression in liver tissues of PBC mice. Due to the limitation of time and funds, we did not examine the effect of GRK2 on ENDRB in in liver tissues of PBC mice. This is a limitation of our study.

The activation of NF-κB signaling pathway has been reported to be correlated with ENDRB functions and expression^32–34^. For instance, the introduction of IKK (IκB kinase) inhibitors weakened ENDRB-mediated contraction^35^. Also, GRK2 was involved in the regulation of NF-κB signaling pathway^36,37^. For instance, GRK2 inhibitor paroxetine markedly abated arginine vasopressin-evoked NF-κB activation in neonatal rat cardiac fibroblasts^37^. Previous studies have showed that NF-κB signaling pathway is implicated in PBC pathogenesis^38,39^. For instance, sirtuin-1 activation alleviated liver injury, reduced serum AST and ALT levels, inhibited pro-inflammatory cytokines secretion in poly I:C-induced PBC mouse models by inhibiting NF-κB signaling pathway^40^. Thus, we further explored whether ENDRB could influence PBC progression by regulating NF-κB pathway. Our data disclosed that ENDRB overexpression or agonist activated NF-κB pathway, while ENDRB knockdown or blockade inhibited NF-κB pathway in liver tissues of PBC mice.

In conclusion, EDNRB and EDN2 expression levels were notably up-regulated in liver tissues and serum samples of PBC patients and mice. EDNRB overexpression or activation enhanced liver injury and pro-inflammatory responses and accelerated PBC progression by up-regulating GRK2 and activating the NF-κB pathway in mice. EDNRB depletion or inhibition could notably ameliorate PBC-mediated liver injury and inflammation in mice, suggesting the potential therapeutic value of EDNRB inhibitor for PBC. This is the first study to elucidate the vital roles of EDNRB in PBC-related liver injury and inflammation. Moreover, we demonstrated that GRK2 and NF-κB pathway were involved in the modulation of EDNRB function in PBC.

## Supplementary Information

Supplementary Information.

## Data Availability

The datasets used and/or analysed during the current study are available from the corresponding author on reasonable request.

## Abbreviations

PBC: Primary biliary cholangitis
AMA: Antimitochondrial antibody
UDCA: Ursodeo-xycholic acid
EDN1: Endothelin-1
EDN2: Endothelin-2
EDNRA: Endothelin receptors A
EDNRB: Endothelin receptors B
ALT: Alanine transaminase
ALP: Alkaline phosphatase
AST: Aspartate transaminase
GGT: γ-Glutamyltranspetidase
IFN-γ: Interferon gamma
TNFα: Tumor-necrosis factor alpha
IHC: Immunohistochemical
Ad-shEDNRB: Adenoviruses carrying EDNRB knockdown fragment
Ad-EDNRB: EDNRB full-length CDS
GPCRs: G protein-coupled receptors
GRK2: G protein-coupled receptor kinase 2
