# Supplementary material for: Endothelin receptor B enhances liver injury and pro-inflammatory responses by increasing G-protein-coupled receptor kinase-2 expression in primary biliary cholangitis
Source: Sci Rep. 2022 Nov 17;12:19772. doi: 10.1038/s41598-022-21816-x (PMC9672122; doi:10.1038/s41598-022-21816-x)

**Endothelin receptor B enhances liver injury and pro-inflammatory responses by increasing G-protein-coupled receptor kinase-2 expression in primary biliary cholangitis**

**Guoxin Xu^1a^, Yanping Gong^2a^, Fenying Lu^3^, Bin Wang^3^, Zaixing Yang^4^, Long Chen^1^,**

**Jingyu Min^3^, Cuie Cheng^3^******, Tingwang Jiang^5^***

^1^ Department of Clinical Laboratory, The Affiliated Zhangjiagang Hospital of Soochow University, Zhangjiagang 215600, China.

^2^ Department of Clinical Immunology, Institution of Laboratory Medicine of Changshu, Changshu 215500, China.

^3^ Department of Gastroenterology, the Affiliated Changshu Hospital of Xuzhou Medical University, Suzhou 215501, China.

^4^ Department of Laboratory Medicine, Huangyan Hospital of Wenzhou Medical University, Taizhou First People’s Hospital, Taizhou 318020, China.

^5^ Department of Key Laboratory, The Affiliated Changshu Hospital of Xuzhou Medical University, Changshu 215500, China.

^a^ These authors contributed equally to this work and should be considered co-first authors

*Corresponding author: Tingwang Jiang, Email: jtwgyp@163.com.

**Address:** Department of Key Laboratory, The Affiliated Changshu Hospital of Xuzhou Medical University, Changshu 215500, China.

**Co-corresponding author: Cuie Cheng, Email: Cuie_cheng@163.com.

**Address:** Department of Gastroenterology, the Affiliated Changshu Hospital of Xuzhou Medical University, Suzhou 215501, China.

**Images of the original blots**

**Figure 2F**

EDN1


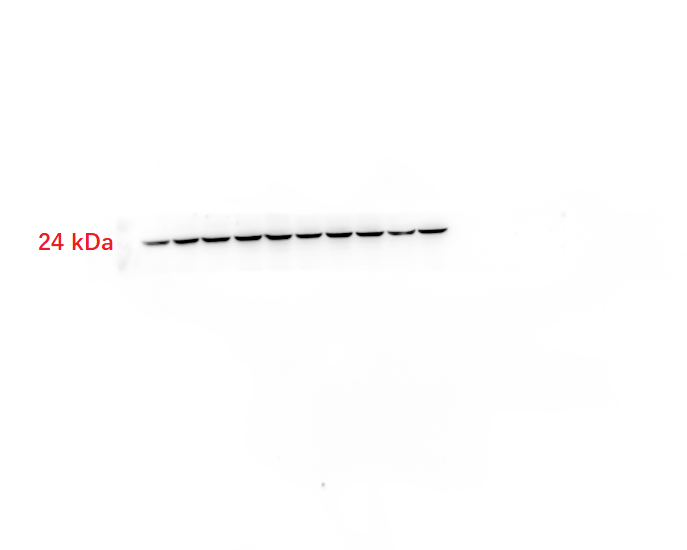


EDNRA


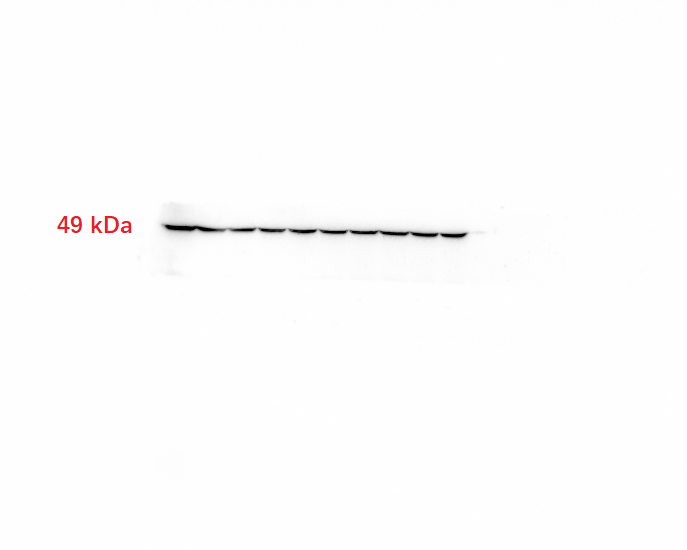


EDN2


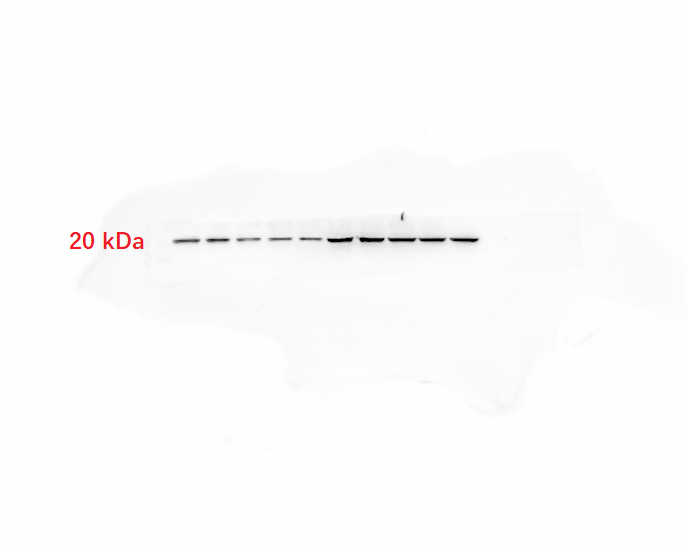


EDNRB


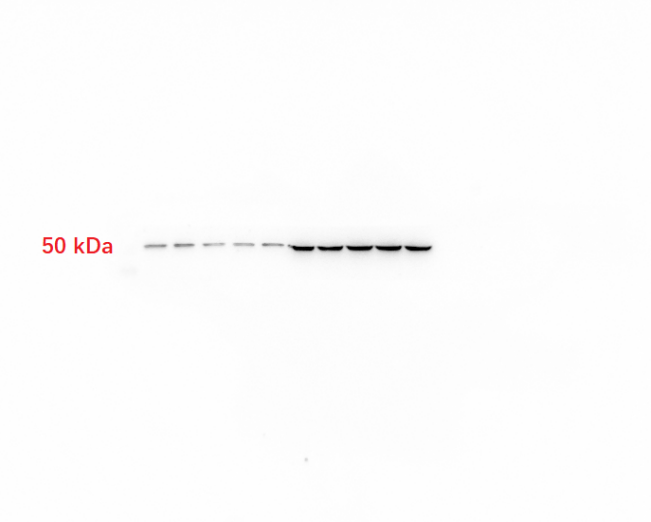


β-actin


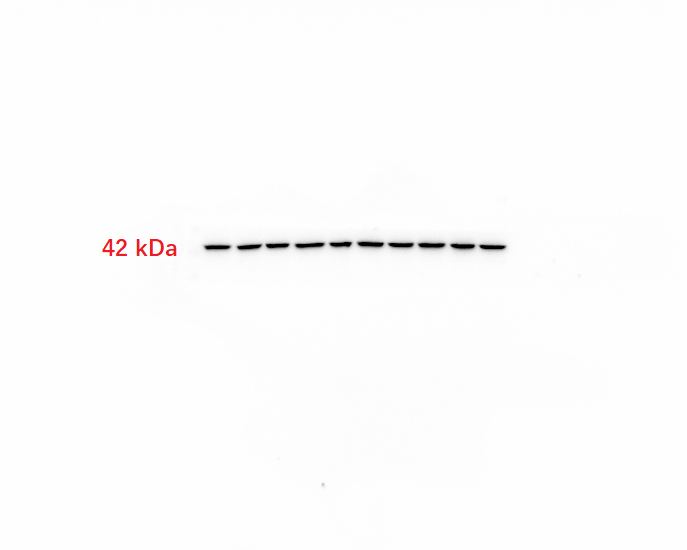


**Figure 2G**

EDN1


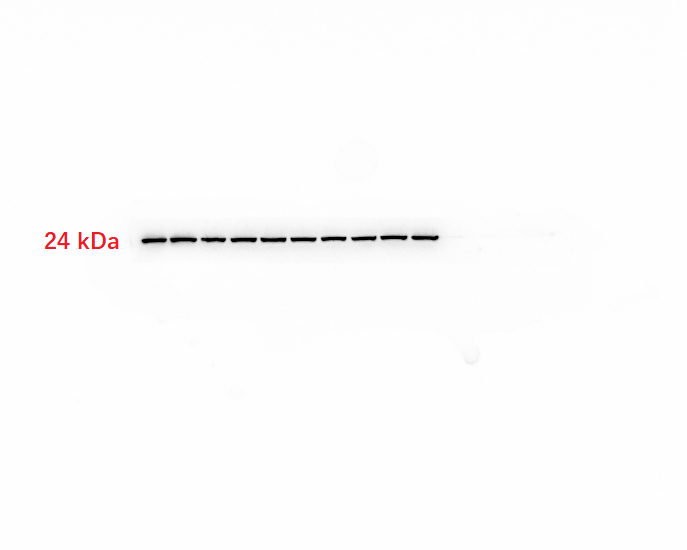


EDN2


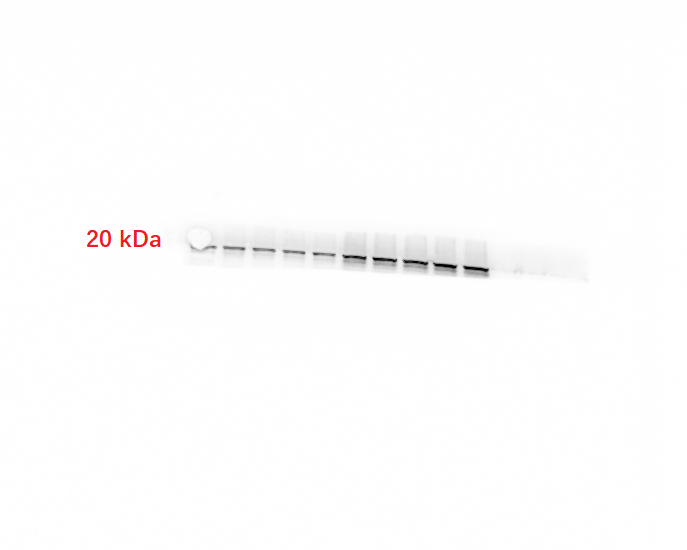


β-actin


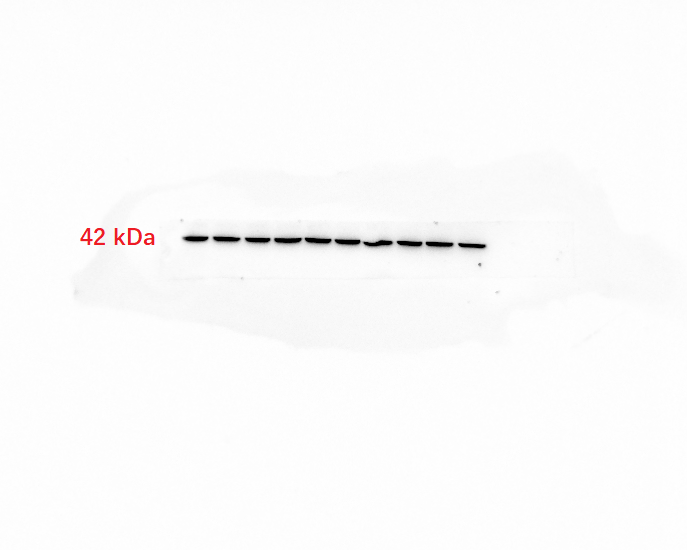


**Figure 4D**

EDN1


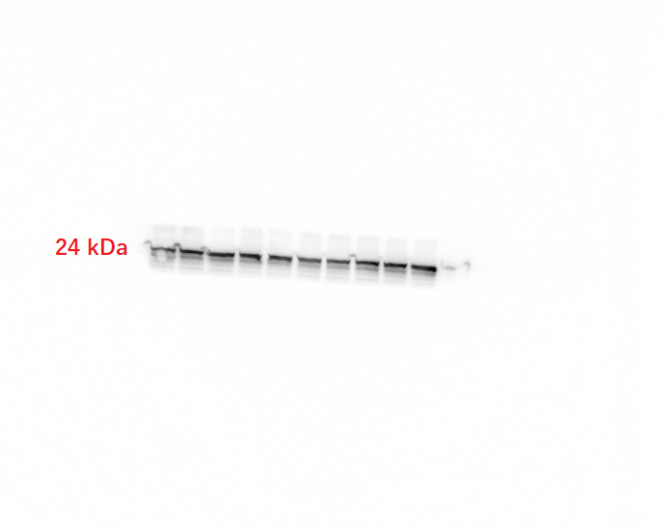


EDN2


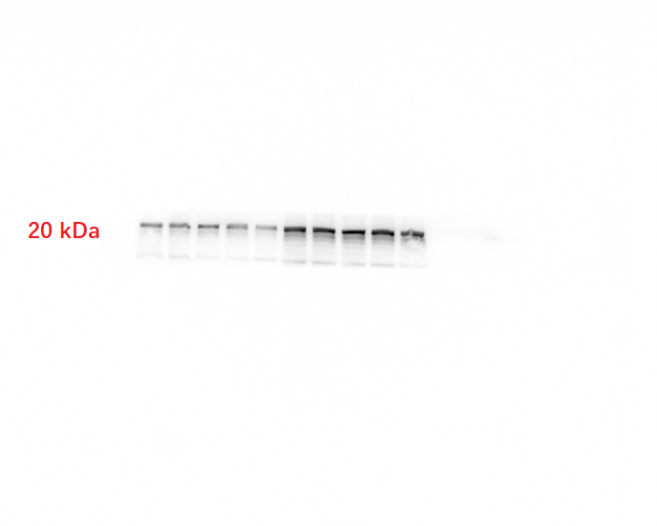


β-actin


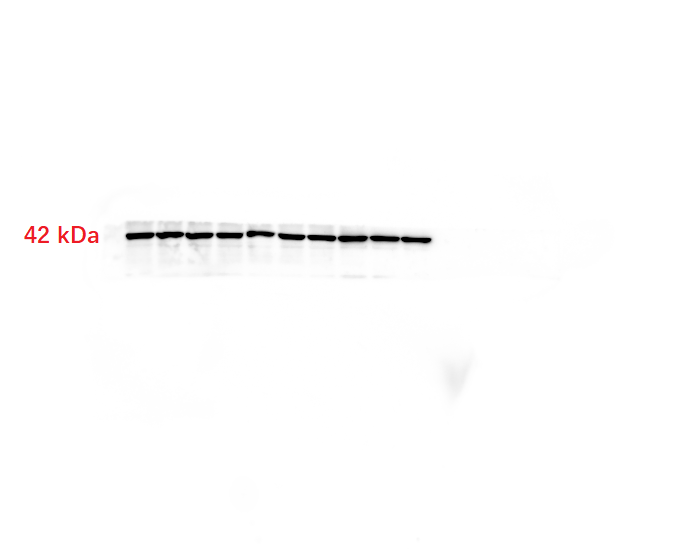


**Figure 4E**

EDN1


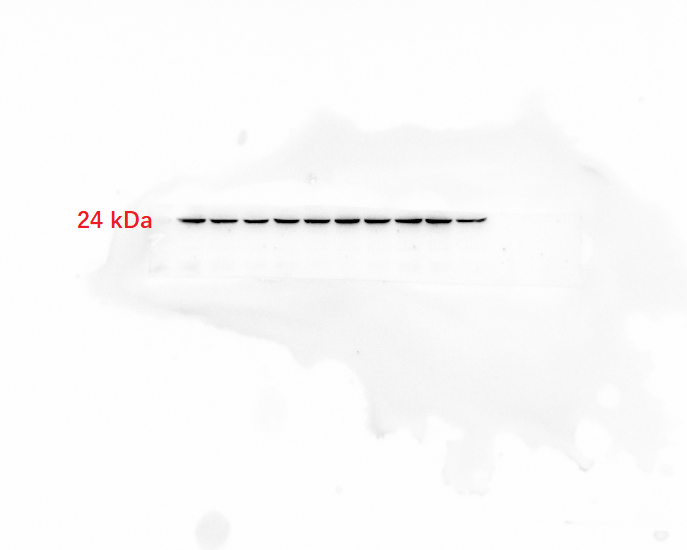


EDNRA


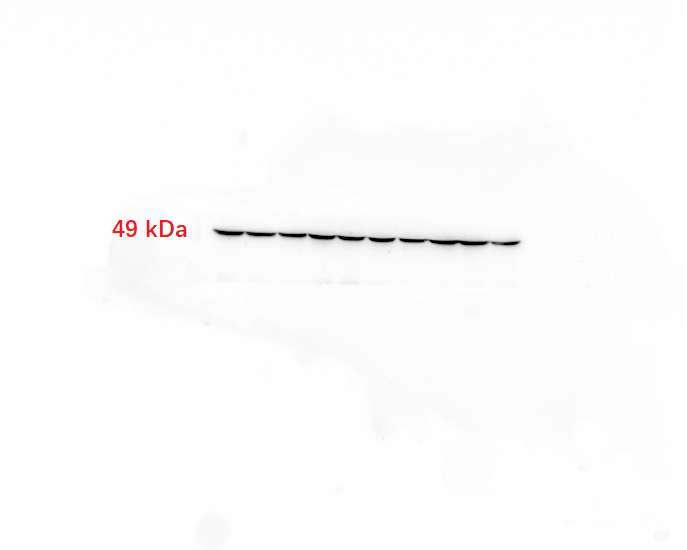


EDN2


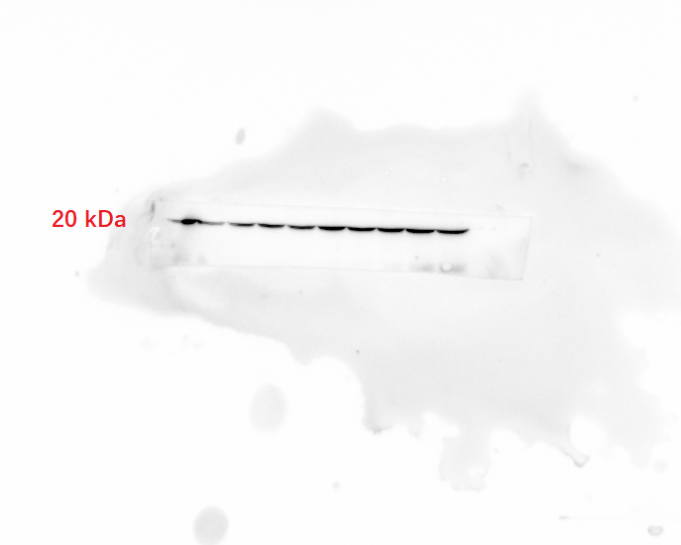


EDNRB


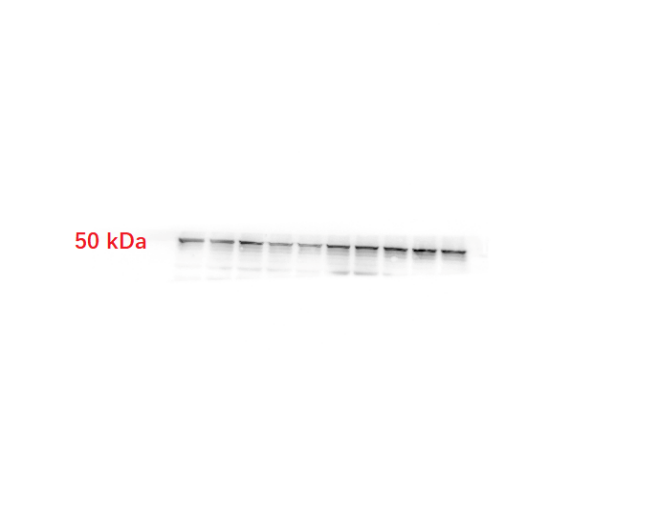


β-actin


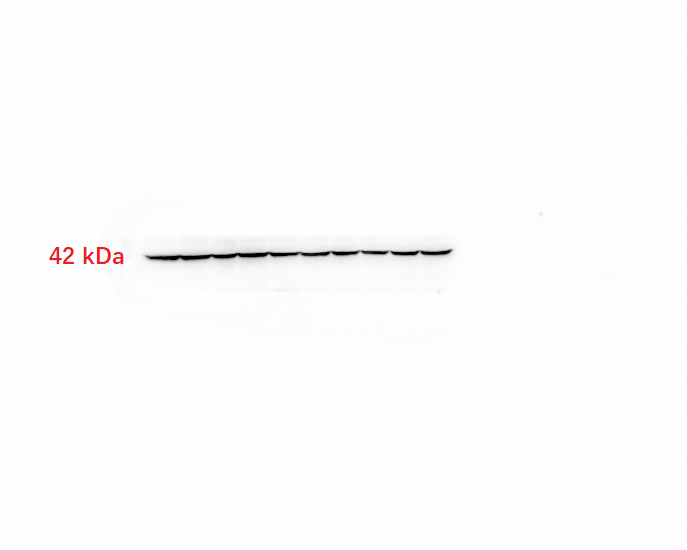


**Figure 5D**

EDN1


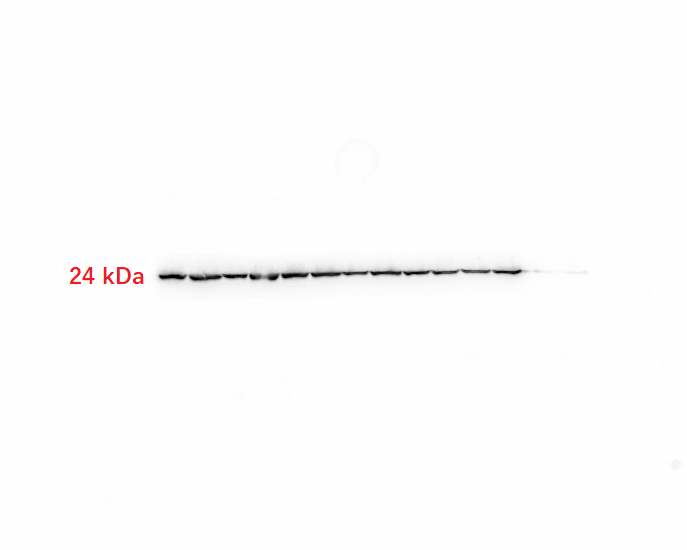


EDN2


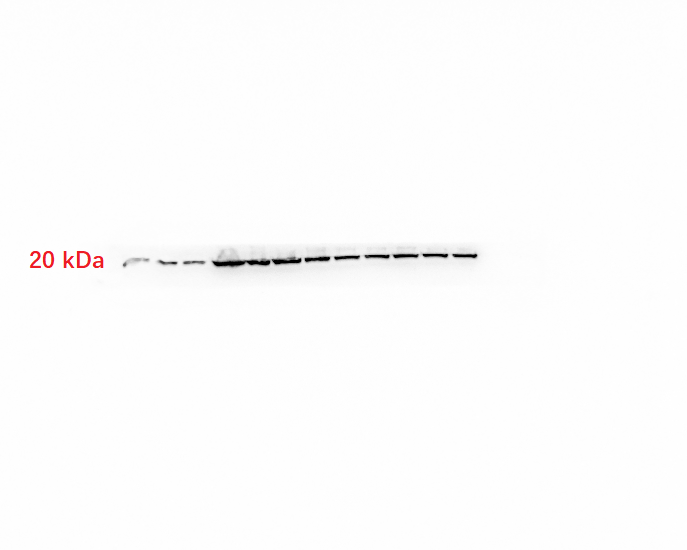


β-actin


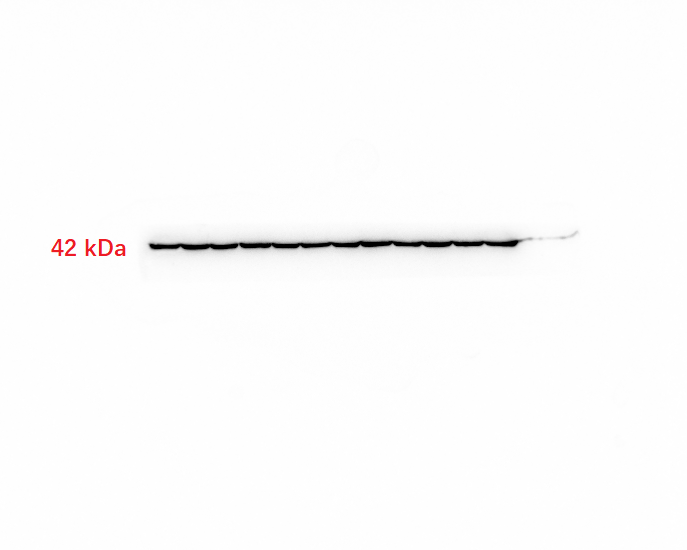


**Figure 5E**

EDN1


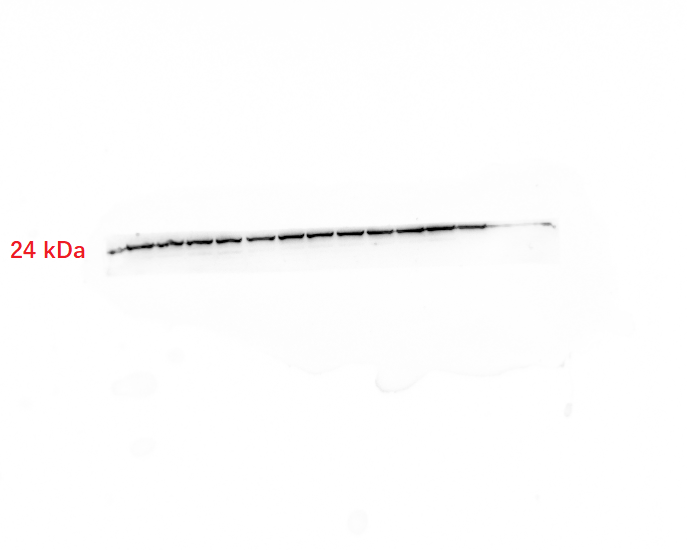


EDNRA


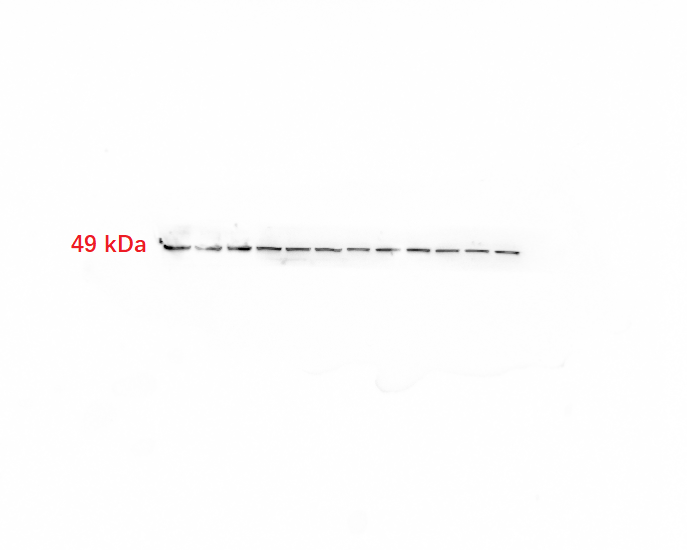


EDN2


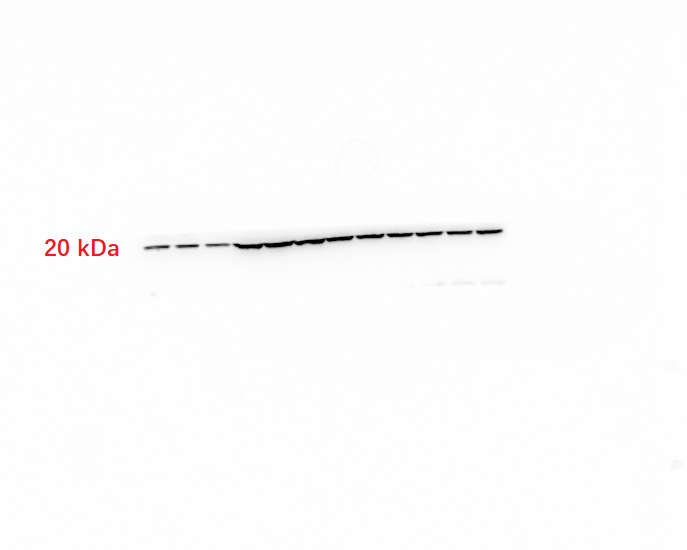


EDNRB


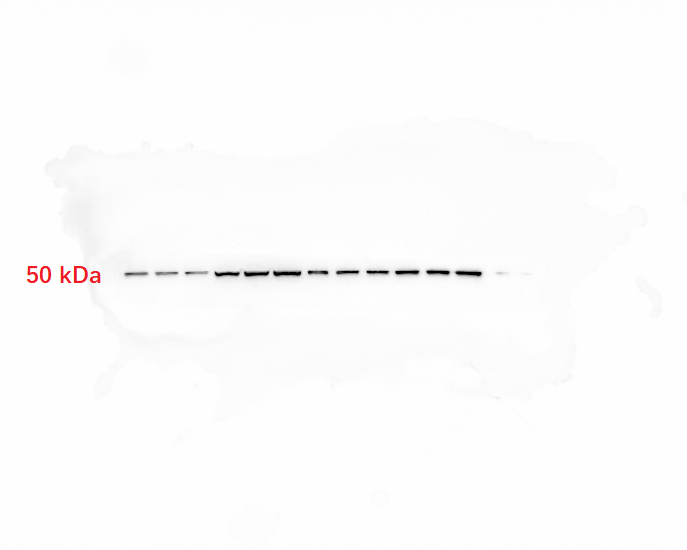


β-actin


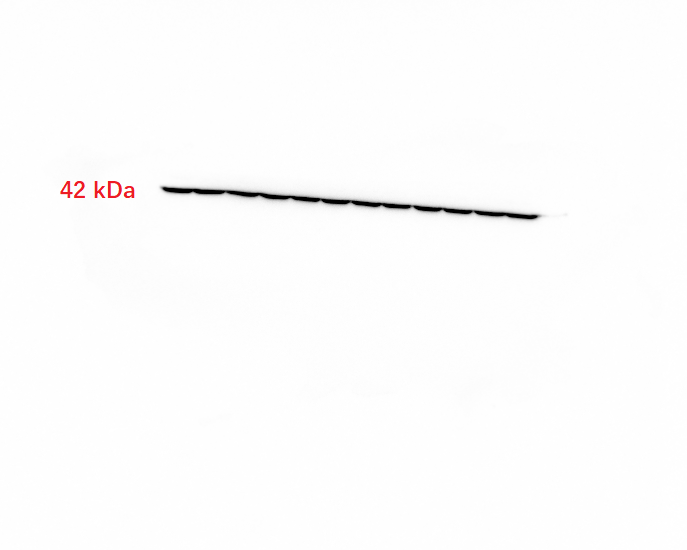


**Figure 6G**

EDNRB


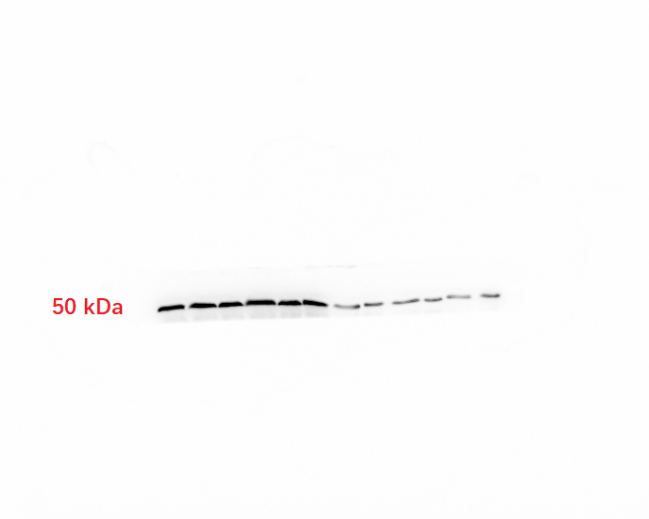


GRK2


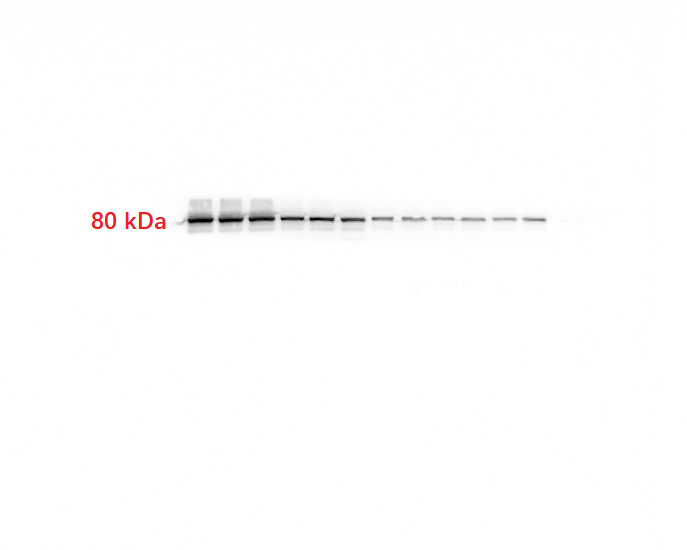


IKKα


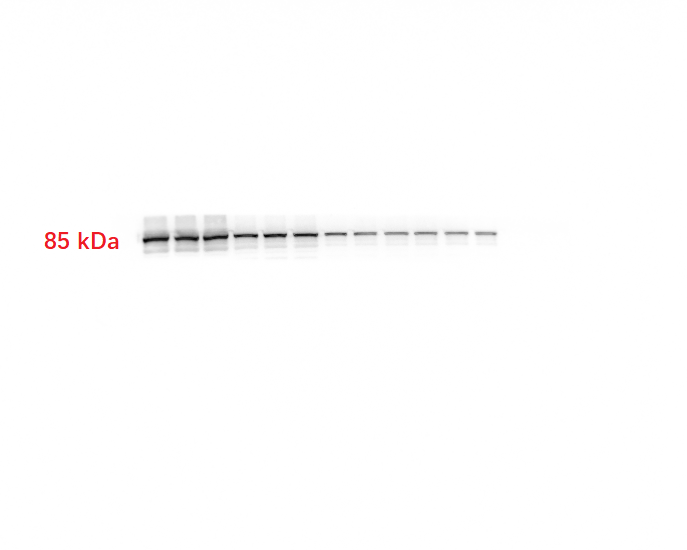


p- IKKα


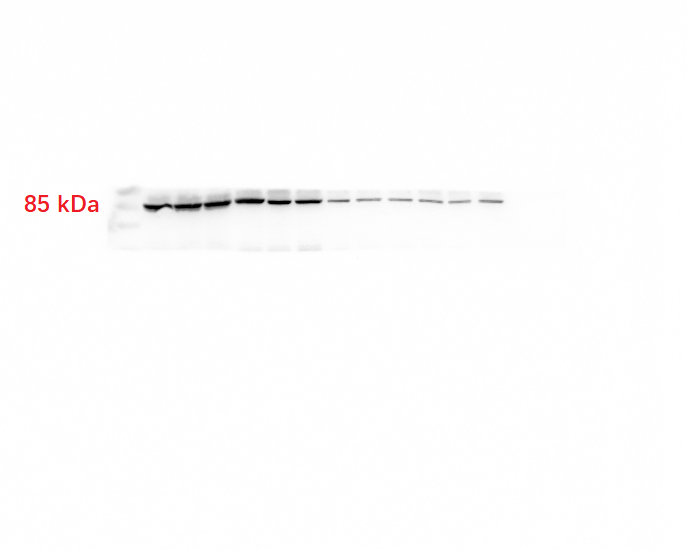


IKKβ


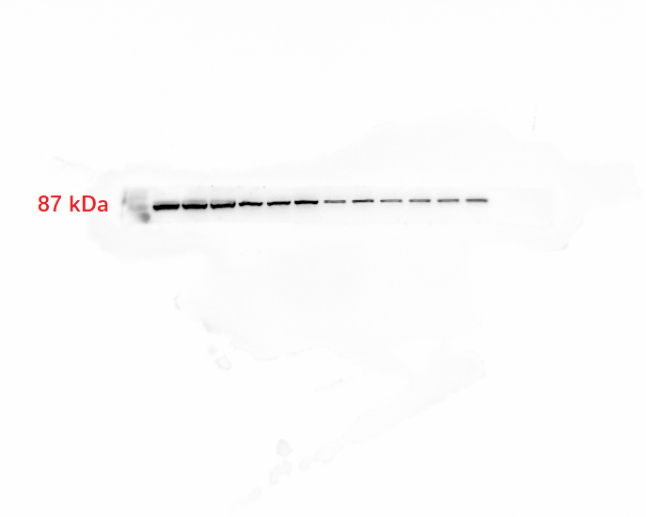


p- IKKβ


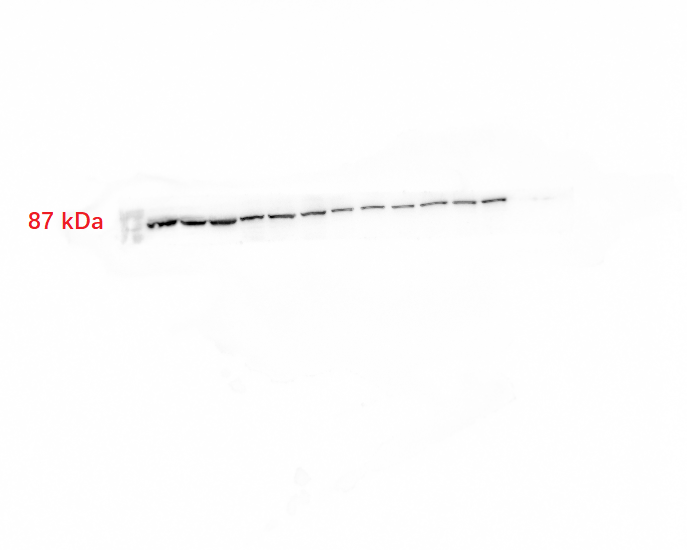


NF-κB


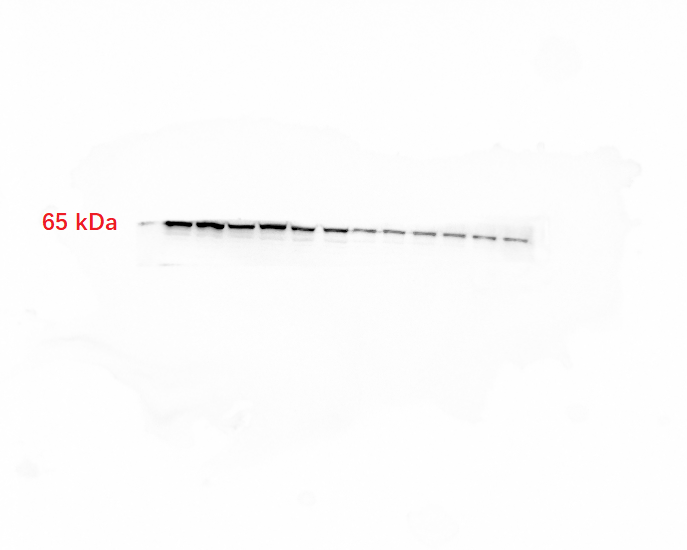


p- NF-κB


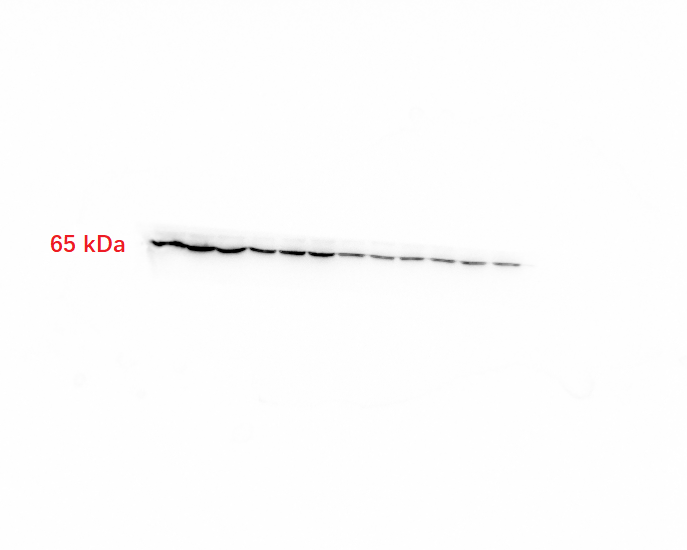


β-actin


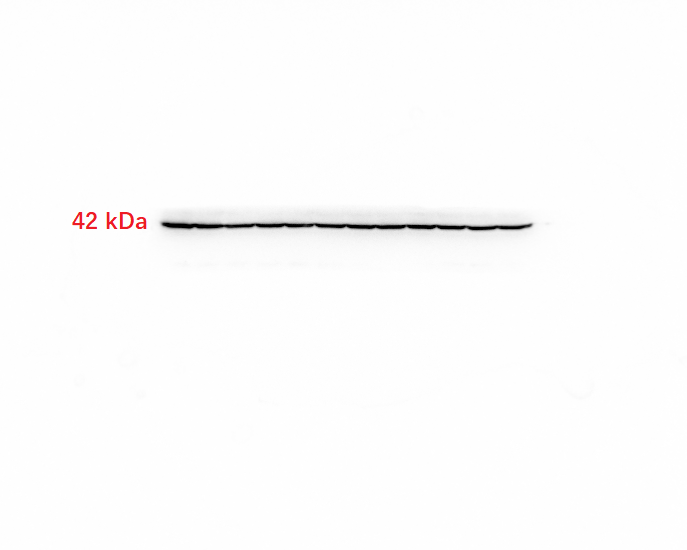


**Figure 7G**

EDNRB


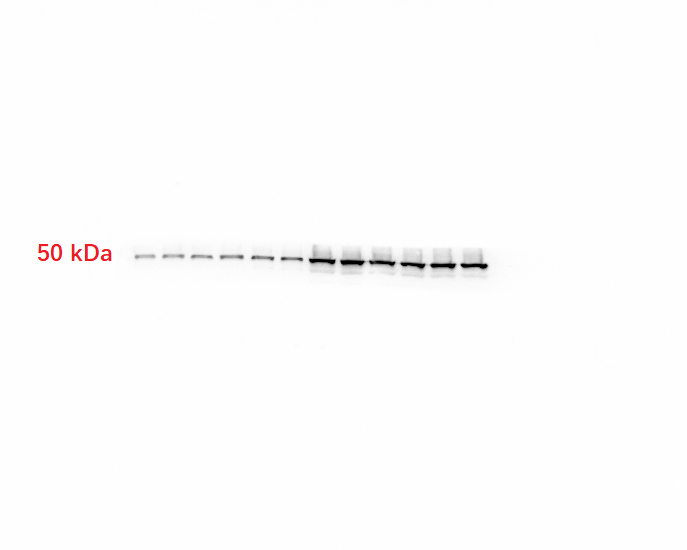


GRK2


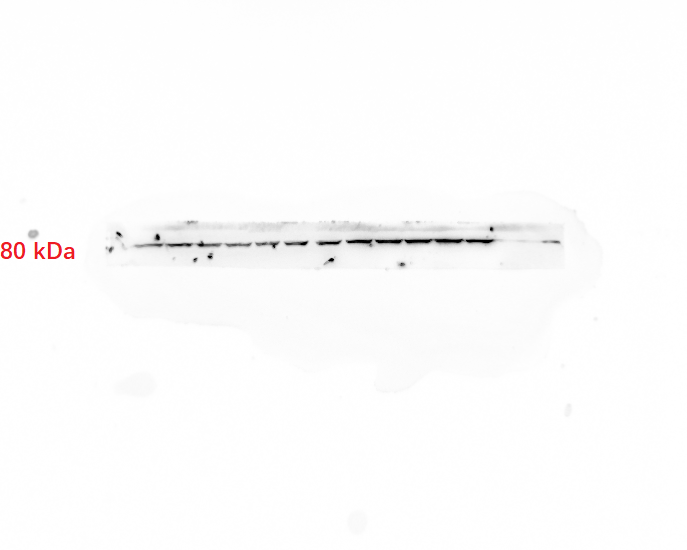


IKKα


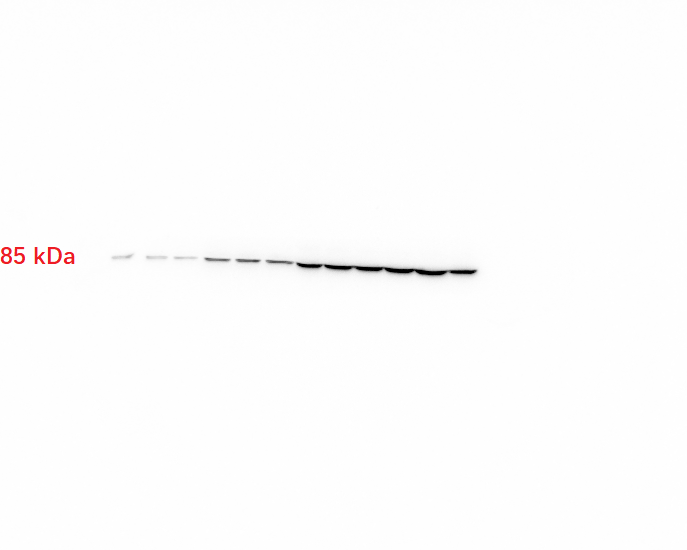


p-IKKα


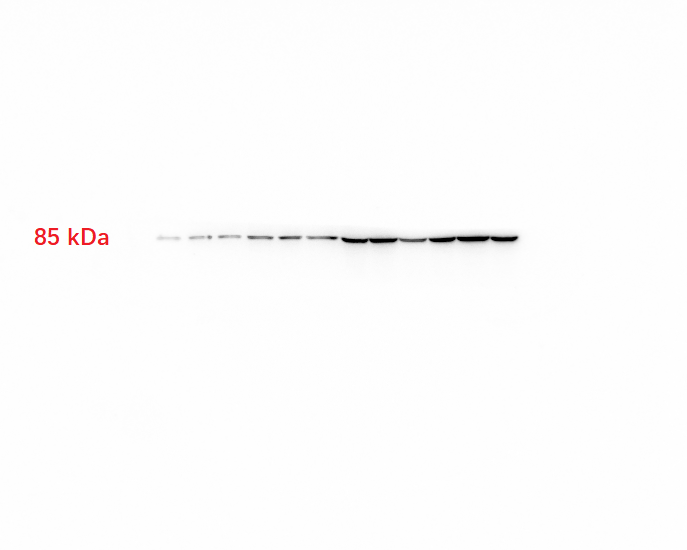


IKKβ


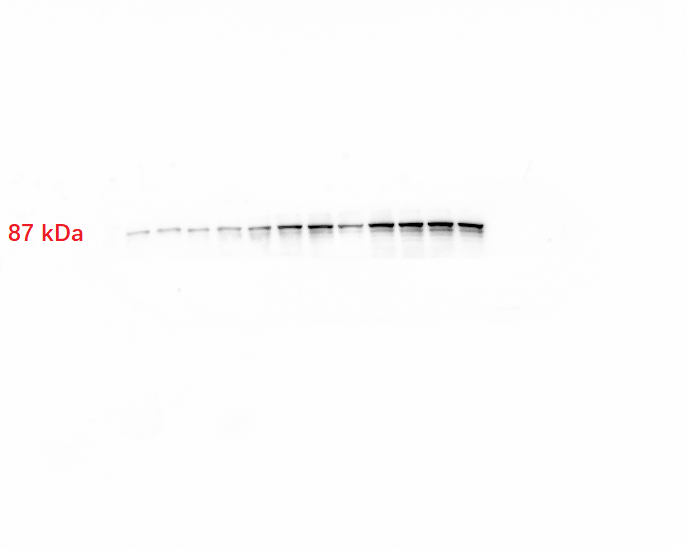


p-IKKβ


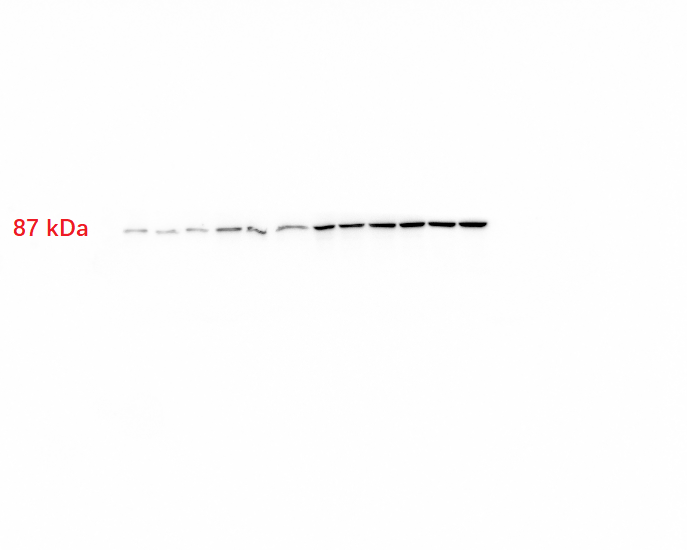


NF-κB


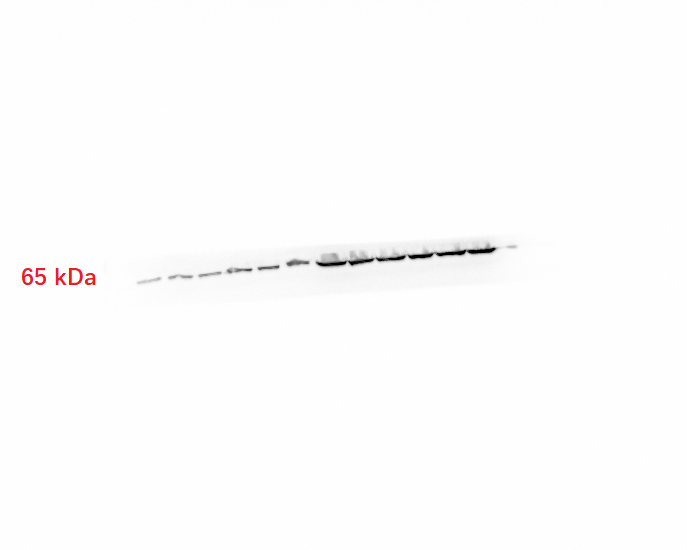


p-NF-κB


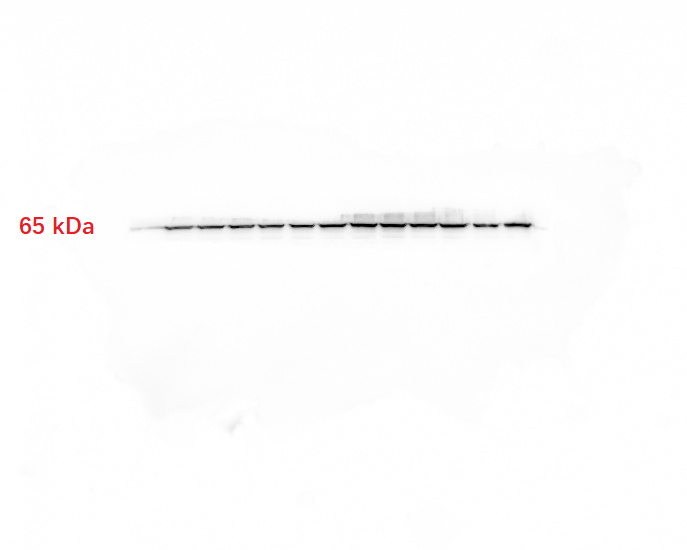


β-actin


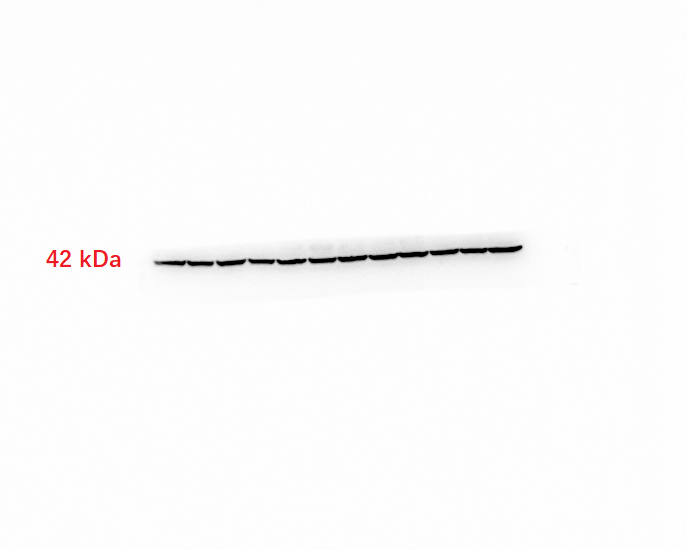

Supplement: Supplementary file 1 — Supplementary Information. [file 41598_2022_21816_MOESM1_ESM.docx]
